# Scrotal circumference: A predictor of testosterone concentration and certain attributes of seminal vesicles influencing buffalo male fertility

**DOI:** 10.14202/vetworld.2018.739-747

**Published:** 2018-06-05

**Authors:** S. Mahmood, A. Kumar, R. Singh, M. Sarkar, G. Singh, M. R. Verma, G. V. P. P. S. R. Kumar

**Affiliations:** 1 Division of Animal Reproduction, ICAR-Indian Veterinary Research Institute, Izzatnagar - 243 122, Bareilly, Uttar Pradesh, India; 2 Division of Biochemistry, ICAR-Indian Veterinary Research Institute, Izzatnagar - 243 122, Bareilly, Uttar Pradesh, India; 3 Division of Pathology, ICAR-Indian Veterinary Research Institute, Izzatnagar - 243 122, Bareilly, Uttar Pradesh, India; 4 Division of Physiology and Climatology, ICAR-Indian Veterinary Research Institute, Izzatnagar - 243 122, Bareilly, Uttar Pradesh, India; 5 Division of Livestock Economics and Statistics, ICAR-Indian Veterinary Research Institute, Izzatnagar - 243 122, Bareilly, Uttar Pradesh, India; 6 Division of Biotechnology, ICAR-Indian Veterinary Research Institute, Izzatnagar - 243 122, Bareilly, Uttar Pradesh, India

**Keywords:** male buffalo, morphology, scrotal circumference, seminal vesicles, sequencing, testosterone

## Abstract

**Aim:**

The aim of this study was to evaluate the relationship of scrotal circumference (SC) with plasma testosterone, seminal vesicles (SVs) weight, and its secretion as measurable indicators of fertility and also to sequence and establish phylogenetic relatedness of certain SV protein genes with other species as such integrated approach is lacking.

**Materials and Methods:**

Altogether, 59 apparently healthy male buffaloes sacrificed at slaughterhouse were selected (irrespective of breed) for measuring SC and collecting blood and paired SVs. The SC was measured at greater curvature using soft thread. In the present study, blood plasma testosterone, cholesterol, protein, and glucose in addition to SV fructose, citric acid and proteins in SV fluid were also estimated. The SV tissue was fixed in RNAlater for RNA extraction. Male buffaloes were categorized as per total SV weight into Group I (<5.0 *g*), Group II (5.0-7.84 *g*), and Group III (>8.0 *g*) and dentitions-I (≤18 months), II (18-24 months), and III (≥24 months) to assess the effect of weight and dentition age on SC, SV weight, and its certain secretions. Data were analyzed using linear model procedure including Tukey HSD test and Pearson’s correlation coefficient. Variance inflation and condition index were also used to assess multicollinearity.

**Results:**

Gross and histomorphological evaluation of SVs did not show any abnormality. Macronutrients (plasma protein, glucose, and cholesterol) showed non-significant (p>0.05) variation between groups. The SC and SV weight varied significantly (p<0.05) with a significant positive relationship with plasma testosterone, SV protein, fructose, and citric acid. In addition, testosterone concentration also showed increasing trend from Groups I to III but increased significantly (p<0.05) from Group II to III with positive and significant correlations with SV protein, fructose, and citric acid similar to SV weight and SC. Binders of sperm protein (BSP1, 3, and 5) genes (full length) were sequenced and established an evolutionary relationship which is lacking in buffalo.

**Conclusion:**

The present findings established a significant positive correlation of SC with that of other fertility parameters related to SVs weight and its secretions: Fructose, citric acid, and protein (inclusive of BSPs sequenced full length), and testosterone. Therefore, the present integrated approach along with certain semen quality attributes reflecting epididymis function could be used as a predictive fertility marker for grading and selection of breeding bulls and their progenies to develop outstanding bull mother farm.

## Introduction

Scrotal circumference (SC) in *Bos indicus, Bubalus bubalis*, and *Bos taurus* is variable depending on body weight and age. In cattle irrespective of breed, the standard of SC for mature and >2 years of bulls was reported as 30 cm and 32 cm, respectively [1], while in buffalo, SC has been reported to be 40% less compared to European breeds of the same age [2]. Optimum testosterone concentration with larger sized SC and SVs plays an important role in enhancing the bull productivity of spermatozoa (12-17 million) per gram testicular tissue [3] and semen quality in Murrah buffalo bulls [2]. Furthermore, relationship across gender suggests that certain genes may affect some critical reproductive mechanisms in both males and females [4] as reproductive organ size (testis and SVs) is an indicator of fertility and marker of the timing of puberty [5]. Interestingly, testis growth is very rapid and almost linear [6] with 0.06-0.07cm/day increase in SC in cattle bulls [7]. Thus, calfhood nutrition is important for larger SC, testes, and early spermatogenesis at 1year of age [8] as larger SC was reported correlated with testosterone in buffalo [9] and age at puberty [10]. Prepubertal testosterone influences the growth of the testes with an optimum number of Sertoli and Leydig cells and synthesis of SV basic proteins [11]. In normal young bull calves, fructose appears in the SVs between 1*^st^* and 3*^rd^* months of life, while in buffalo, one report showed increasing trend of fructose and citric acid in SV fluid with increasing SVs weight [12]. In addition, Serrano [13] evolved evolutionary relationship of buffalo binders of sperm protein 3 (BSP3) (seminal plasma protein A3[SPA3] and partial coding DNA sequence [CDS]). In cattle, Jois *et al*. [14] indicated closer identity between BSP1 and 3 compared to BSP5. However, in buffalo, full-length BSP 1, 3, and 5 gene sequences and phylogenetic evaluation with other species are lacking.

Selection of outstanding sire is a prerequisite to enhance the economic potential of their progenies as bull fertility differences are paramount on herd genetics under optimum nutrition and management. Seminal vesicles (SVs), that add volume, nutrients, and buffers to the semen, are the major accessory sex glands of ruminants.

The objective of the present study was to evaluate the relationship of SC with plasma testosterone, SV weights, and SV secretion (protein, fructose, and citric acid) as predictive measurable indicators of fertility for grading and selection of breeding bulls/their progenies and also to sequence and establish phylogenetic relatedness of certain SV protein genes with other species as such integrated approach is lacking particularly in male buffaloes. We hypothesized that increasing SC could predict the testosterone level, SV weight, and its function as per age.

## Materials and Methods

### Ethical approval

Male buffaloes sacrificed at the local abattoir (government approved) were used for observation/sample collection. There are no ethical issues in this study.

### Study area

Bareilly is a prominent city located at 28°20′49′N, 79°25′18′E, and altitude of 179 *m* above sea level in Northern India with extreme temperature (4°C-44°C) and mean annual rainfall of 1714 *mm*. The rainy season starts in June and extends up to September with humid and warm condition.

### Experimental animals and design

SVs (59 pairs) along with ampullae were dissected out (within an hour) from sacrificed apparently healthy male buffaloes of varying age (as per dentition), irrespective of breed and nutritional status, at slaughterhouse, Bareilly (Uttar Pradesh, India). On the basis of total SV weight and dentition age, SVs were categorized into SV weight Group I (<5.0 *g*), Group II (5.0-7.84 *g*), and Group III (>8.0 *g*) and dentitions-I (≤18 months, milk teeth), -II (18-24 months, one pair permanent), and -III (≥24 months, > 2 pair) groups, respectively. Blood was also collected in tubes with oxalated fluoride and heparin sodium (SRL, India) anticoagulants, respectively, for the estimation of glucose, protein, cholesterol, and plasma testosterone. The SC was measured at greater curvature using soft thread. Age was predicted as per dentition formula. The SVs were freed from adhering tissues, then placed in a sealing polythene bag, and finally brought to laboratory over ice in thermos.

### Processing of samples

In the laboratory, the SVs were processed [15] and weighed using digital balance (CX220, Citizon) and then was flushed [15]. Blood plasma and SV flushed fluid were centrifuged at 3000 rpm for 15 min. Small fragments (more or less from the same location and side) from each SV were also fixed in 10% buffered formalin for histomorphological studies using hematoxylin and eosin (*H and E*) stained sections. Blood plasma, flushed SV fluid, and RNAlater (Qiagen, Germany) fixed SVs tissues were kept at −20°C until processing.

### Total RNA extraction

About 40-100 mg tissue was weighed and transferred in pestle and mortar (treated with 0.1% DEPC [Amresco] for 24 h and autoclaved) containing about 5-7 ml LN2 as chilling and hardening agent and grinded to near powder and then triturated in 1.0 ml Trizol and then rewashed with 0.5 ml more Trizol making the final volume of 1.5 ml Trizol (BR Biochem) and pipetted into 2.0 ml RNAase free (Eppendorf) tube (0.1% DEPC treated). To this, 0.3 ml chloroform (0.2 ml/1.0 ml Trizol) was added and allowed to stand with intermittent mixing for 10 min and centrifuged at 12,000 rpm for 15 min at 4°C (SIGMA 3-18K). From this, pipetted out constant volume (0.7 ml) of supernatant in 1.5 ml conical microcentrifuge tube and into it added 0.75 ml isopropyl alcohol (BR Biochem, 0.5 ml/1.0 ml Trizol) to precipitate RNA, allowed to stand with intermittent upside downmixing for 10 min at room temperature (23-25°C) and centrifuged as above. The supernatant was carefully decanted, and pellet was washed with 1.5 ml (1.0 ml/1.0 ml Trizol) 85% ethanol (BR Biochem) and finally centrifuged at 12,000 rpm for 5 min at 4°C. Ethanol was decanted with slow tapping on tissue paper. Finally, pellet was dissolved in 20-30 µl nuclease free water (Qiagen, Germany). RNA concentration (ng/µl) and purity (OD260/OD280 ratio) were estimated using Eppendorf spectrometer.

### cDNA synthesis and amplification of buffalo BSP gene

RNA was reverse transcribed into cDNA with reverse transcriptase kit (Qiagen, Germany). Full-length sequence of *B. taurus* (cattle) BSP1, BSP3, and BSP5 gene CDS with accession number NM_001001145.1, NM_174840.1, and NM_174842.2, respectively, was used to design respective primers using Gene tool with insertion of restriction site (peT28c) at 5^ı^ end for cloning (Table-1).

**Table-1:** Target genes, primer sequence ((5^’^3) 5 with peT28c vector restriction sites (forward and reverse), amplicon length (bp), and NCBI accession number.

Target gene	Sequence of primers with vector	Amplicon length (bp)	NCBI accession no.
BSP1	For: 5’GATCGAATTCTTATGGCACTGCAGTTGGGGC3	440	NM_001001145.1
	Rev: 5’GATCAAGCTTCTAGCAATACTTCCAAGCTCTGTCCT3’		
BSP3	For: 5’GATCGAATTCATATGGCACTGCGTTTGGGG3’	446	NM_174840.1
	Rev: 5’GATCAAGCTTCTAGCAATACTTCCAAACTCCATCCT3’		
BSP5	For: 5’GATCGAATTCATATGGCACCGCTAGTGGGGC3’	575	NM_174842.2
	Rev: 5’GATCCTCGAGCTAGCAATACTTCCAAGCTTTATCCC 3’		

BSP=Binders of sperm protein

### Polymerase chain reaction (PCR) amplification

PCR amplification was run in 25 µl reaction mixture containing cDNA 0.6 µl, 10× PCR buffer 2.5 µl; forward and reverse primer each 0.5 µl; dNTPs mix (10 mM each) 0.5 µl; Taq DNA Polymerase 0.3 µl (5U/µl) and milliQ water 20.1 µl for each genes. The PCR protocol included initial denaturation at 95°C for 5 min (BSP1 and BSP3), while 94°C for 4 min for BSP5; 35 cycles for BSP1 and BSP3 but 30 cycles for BSP5, denaturation (95°C 30 s for BSP1 and BSP3 but 94°C for 1 min for BSP5), annealing at 58°C for 30 s, 54°C for 20 s, and 54°C for 30 s, respectively, for BSP1, BSP3, and BSP5, and extension (72°C for 45 s for BSP1 and 30 s for BSP3 and BSP5), followed by one cycle of final extension at 72°C for 7 min (BSP1 and BSP3) but 5 min for BSP5 gene. The PCR product was electrophoresed in 1% agarose gel at 100 mv for 45 min, and bands were visualized in Gel documentation system (GELDOC™XR^+^BIO-RAD).

**Figure-1:**
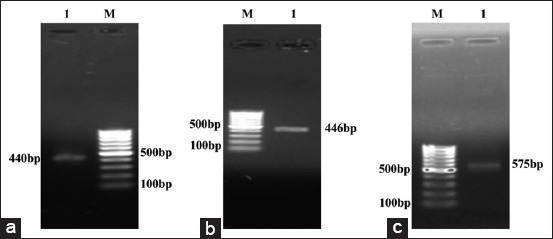
Agarose (1%) gel depicting PCR amplified purified buffalo BSP1 (Fig.1a), BSP3 (Fig.1b) and BSP5 (Fig.1c) with vector sequence. Lane 1: PCR product. Lane M: 100bp DNA ladder.

Specific PCR product band at desired position was dissected out from agarose gel and was purified using Gel purification Kit (Geneaid, BR Biochem). DNA was quantified (BSP 1=16.1 ng/µl, BSP3=17.8 ng/µl, and BSP5=9.0 ng/µl) with purity ratio (OD260/OD280) of 1.90, 1.93, and 1.97, respectively, in Nanodrop, ND-1000 spectrophotometer. Purified and quantified DNA was electrophoresed in 1% agarose gel at 100 mv for 45 min (Figures-1a-c).

### Sequencing of gene

Purified PCR products were got sequenced using Sangers Dideoxy Sequencing Technology (Eurofins Genomics India Pvt. Ltd., Bengaluru, India). The sequence obtained was subjected to blast (http://blast.ncbi.nlm.nih.gov/Blast.cgi) to retrieve BSP1, BSP3, and BSP5 sequences of other species. Finally, the full-length sequences were submitted to GenBank as buffalo BSP1, BSP3, and BSP5 with accession number as KR703587, KR814819, and KR869821 with open reading frame (ORF) of 417 bp (138 amino acid), 423 bp (140 amino acid), and 552 bp (183 amino acid) nucleotide sequence, respectively. The nucleotide sequence of buffalo BSP1, BSP3, and BSP5 was aligned and compared with other species reference sequences available in GenBank. Initially, the nucleotide sequences of buffalo BSP1, BSP3, and BSP5 were subjected to multiple sequence alignment (Clustal W) followed by fit model selection for DNA and finally phylogeny reconstruction (maximum likelihood bootstrapping: 1000 replications) using MEGA7.

### Estimations

- Plasma testosterone was estimated using RIA testosterone, direct kit (Beckman Coulter, Immunotech, Prague10, Czech Republic).
- Plasma glucose, cholesterol, protein, and SV protein were estimated using standard kits (Autospan, Surat, India).
- In SVs, fluid citric acid and fructose concentration were estimated by colorimetric method [12] with certain modifications. The optical density (OD) value was plotted against the different concentration in Excel program to obtain standard curve with formula for citric acid (Y=0.762x) and fructose (Y=0.669x+0.011) where Y=OD and x=concentration. Finally, x value was multiplied by factor 10 to get concentration (mg/ml) of citric acid and fructose, respectively, in SV flushed fluid.

Citric acid (Merck, India) stock solution (10 mg/ml, w/v) was used to prepare as standards of 5 µl (0.05 mg), 10 µl (0.1mg), 20 µl (0.2 mg), 40 µl (0.4 mg), and 80 µl (0.8 mg) in 15 ml centrifuge tube and made it to 100 µl (0.1 ml) by triple distilled water (TDW), while 0.1 ml TDW was taken as control in duplicate. A 1.9 ml of 10 % trichloroacetic acid (W/V) was added into above and then mixed for 5 min to precipitate protein and centrifuged at 2000 rpm for 15 min. Supernatant was taken in separating funnel (100/125). A 1 ml of 15 N sulfuric acid (MW 98.08) was added and mixed for 1 min followed by adding 0.2 ml of 1M potassium bromide (MW-119.00) and mixed for 1 min. Finally, 0.3 ml of 0.3 M potassium permanganate (MW-158.0) was added and thoroughly shaken for 1 min and was allowed to stand for 15 min for reaction to complete. Depending on the concentration, color changes from light-brownish to brownish. Then, the excess of potassium permanganate was neutralized by addition of 0.5 ml of 1.5M sodium nitrite (MW 69.00) and shaken until mixture became colorless. Later to this, 0.5 ml of 2M urea solution (MW-60.06) was added and funnel was shaken until gas formation ceased. Finally, 10 ml petroleum ether (60-80 pp, SRL) was added to extract pentabromo - acetate formed. This was vigorously shaken for 1 min and allowed to stand, later transferred the upper layer in a 30 ml tube, and washed thrice with 3 ml TDW and shaken well for 1 min; each time, bottom layer was discarded. Finally, top layer was mixed with 6 ml sodium sulfide hydrate reagent (MW-78.04) in 40 ml ethylene glycol (AR, SRL, India) and made to 100 ml and shaken for 15 min intermittently. Depending on the concentration, light-yellow to yellow to dark yellow color developed. The OD was also recorded for unknown samples at 445 nm.

Fructose (Merck, India) stock solution (05 mg/ml, w/v)) was used to prepare standards of fructose as 5µl (0.025 mg), 10 µl (0.05 mg), 20 µl (0.1 mg), 40 µl (0.2 mg), 80 µl (0.4 mg) and 160µl (0.8mg) in 15 ml test tube in duplicate and made to 3 ml by TDW, while 3.0 ml TDW was taken as control in duplicate. Into these tubes, added 0.5 ml of 0.3N barium hydroxide (MW-315.45, filtered by Whatman no.1) and 0.5 ml of 5 % zinc sulfate (MW-287.56), finally mixed and centrifuged at 2000 rpm for 15 min. From this, taken 2 ml of supernatant (protein free) fluid in 10 ml test tube and added 02 ml of 0.1% ethanolic solution of resorcinol (MW 110.11). Mixed thoroughly and added 6 ml of 30% hydrochloric acid (MW-36.46). After mixing, incubated in water bath, maintained at 90.0°C±1°C for 10 min, and intermittently mixed the solution and cooled immediately. The OD was also recorded for unknown samples using UV-VIS Spectrophotometer (LABINDIA, NF-56) at 540 nm.

### Statistical analysis

To study the effect of total SV weight and dentition age groups on different parameters, general linear model procedure was performed involving Tukey HSD test. To find the association between the SV weight and other parameters, Pearson’s correlation coefficient was performed. In addition, data were also subjected to multiple regression involving variance inflation and condition index to assess multicollinearity and also to derive relationship between variables in unadjusted data. Basic analysis results were expressed as the mean±standard error of the mean (SEM), and differences between groups were considered statistically significant at p<0.05. The complete analysis of data was performed using SAS 9.2 (SAS Institute Inc. 2011. Cary, NC).

## Results

### SV morphology

Findings based on gross and microscopic (H and E stained sections) examination of SVs revealed no any abnormality. While evaluating the glands, various anatomical attributes such as weights in relation to SC and testosterone and pattern of tubuloalveolar ducts were recorded. Grossly, the glands were pale to pink in color with mild to moderately sacculated (unilaterally or bilaterally mostly at the terminal end of the tube) structure resembling the shape of a comma, inverted question mark, and grapes as per SV weight and dentition age.

**Figure-2:**
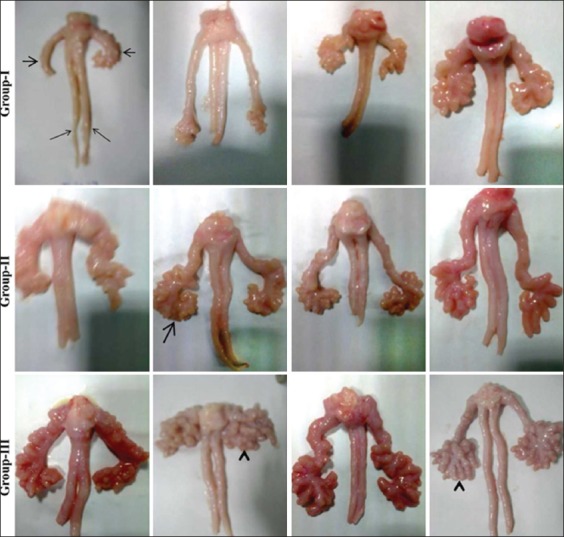
Right and left seminal vesicles (short arrows) showing alveoli arranged in different shapes: Inverted mark (thick arrow), comma (right short arrow) and bunch of grapes (arrow head). The ampullae (thin arrows) are seen in between the SV of buffalo males in all groups as SV weight.

**Figure-3:**
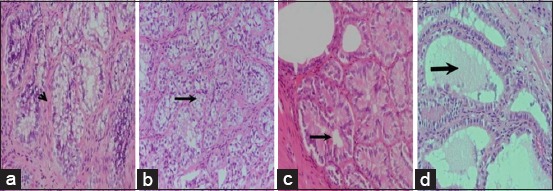
Histomorphological appearance of seminal vesicle of buffalo male showing acini lined with columnar to cuboidal epithelium with vacuolar cytoplasm (thin arrows, Figs. 3b-c) and separated with connective tissue septa (arrow head, Fig. 3a). In developed SV the dilated acinar lumen contained light color secretion (thick arrow, Fig. 3d). H & E. 10X.

The lobulation of tubuloalveolar glands and vascularization was found to increase with SV weight and dentition age. The tubular part was graded as small (31.25, 26.67, and 46.43%, respectively), medium (25.00, 46.67, and 35.71%, respectively), and large (43.75, 26.67, and 17.86%, respectively) in respective SV weight groups. The SV mean weights varied as 3.23±0.27 g (1.389-4.933) in Group I (Figures-2Ia-d); 6.31±0.26 g (5.00-7.84) in Group II (Figures-2IIa-d); and 13.16±0.79 g (8.676-23.52) in Group III (Figures-2IIIa-d). In addition, the tubules were also graded as small (33.33, 37.50, and 50%, respectively), medium (33.3, 41.67, and 25%, respectively), and large (33.33, 20.83, and 25%, respectively) as per dentition age Groups I, II, and III, respectively. The tubular lining epithelial cells, the lamina propria, and muscular layers did not show any abnormality/inflammatory change (Figures-3a-d).

### Assessment of macronutrients status

Mean±SEM of protein, glucose, and cholesterol concentration are presented in Table-2 which showed non-significant variations (p>0.05) indicative of more or less uniform macronutrient status in male buffaloes under the present study. Intraassay coefficient of variation for plasma protein, glucose, and cholesterol was 11.89%, 7.94%, and 9.56%, respectively.

**Table-2:** Mean±SEM plasma protein (g/dl), glucose (mg/dl), and cholesterol (mg/dl) of male buffalo.

Parameters	SV weight Group I	SV weight Group II	SV weight Group III
N	16	15	28
Protein	6.7±0.16^a^	7.01±0.19^a^	6.9±0.16^a^
Glucose	92.1±2.39^a^	103.1±6.12^a^	93.1±3.4^a^
Cholesterol	68.6±3.31^a^	65.96±2.97^a^	64.6±2.57^a^

Same superscripts varied non-significantly. N=Number of observation, SVs=Seminal vesicles, SEM=Standard error of mean

### Relation of SC with testosterone and SV weight

**Figure-4:**
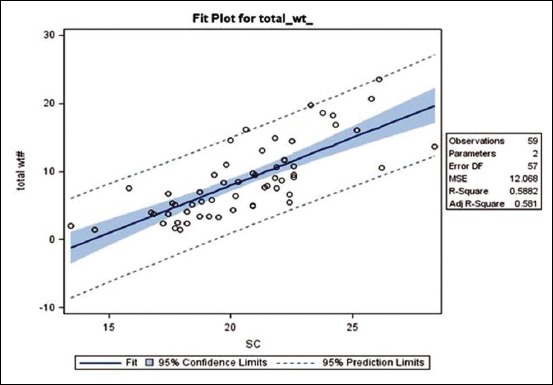
The 95% prediction limits depicting three observations (falling out) as most influential. Linear regression of plot. SV weight on SC shows 1gm increase in total SV weight by 1.395 cm increase in SC (Eq: Total SV weight= -19.96+1.395 SC).

Estimates of SC, plasma testosterone, total SV weight, SV protein, fructose, and citric acid values (Mean±SEM) in three SVs weight groups are presented in Table–3, while fit plot of total SV weight with SC is depicted in Figure-4. Distribution of SC and SVs revealed that 48.15-54.17% and 48.15-62.5% values fall in equal to or greater than mean values, respectively, indicating that data were homogeneous within each dentition age (Table-4). SC values showed significant (p<0.05) increase from SV weight Groups I to III. In addition, SC depicted significant positive relationship with total SV weight (r=0.767), SV protein (r=0.7615), fructose (r=0.6733), citric acid (r=0.4569) and plasma testosterone concentration (r=0.4912). The testosterone concentration was significantly (P < 0.05) higher in SVs weight Group -III compared to Groups-II and I, however, no significant variation was recorded between SVs weight Groups –II and I (Table-3). Similarly, plasma testosterone concentration registered positive and significant correlation with total SV weight (r=0.5574), SV protein (r=0.5402), SV fructose (r=0.4242), and citric acid (r=0.4744). Table-3 also depicts significant (p<0.05) ascending increase in total SV weight from SV weight Groups I-III in accordance with SC change. Furthermore, total SV weight also indicated a significant positive relationship with SV protein (r=0.8893), fructose (r=0.9113), and citric acid (r=0.7365) influencing secretory function of SVs. Interestingly, dentition age groups also recorded ascending pattern of significant (p<0.05) increase in SC and SV weight; however, testosterone also showed increasing pattern with significant (p<0.05) increase from dentition II to III similar to SVs weight groups (Table-5).

**Table-3:** Mean±SEM of scrotal circumference (cm), total seminal vesicle weight (g), and plasma testosterone concentration (ng/ml), SV protein (mg/2 ml, SV fructose (mg/2 ml), and SV citric acid (mg/2 ml) in three SV weight groups in male buffalo.

Parameters	SV weight Group I	SV weight Group II	SV weight Group III
N	16	15	28
SC	17.8±0.48^c^	19.6±0.53^b^	22.6±0.42^a^
Testosterone	0.04±0.01^b^	0.09±0.03^b^	0.29±0.07^a^
Total SV weight	3.03±0.27^c^	6.31±0.26^b^	13.16±0.79^a^
SV proteins	2.94±0.52^c^	7.89±1.23^b^	16.83±1.37^a^
SV fructose	0.16±0.06^b^	1.36±0.36^b^	5.94±0.65^a^
SV citric acid*	0.67±0.16^b^	0.56±0.14^b^	2.89±0.34^a^

* N=15, 14 and 27 respectively. Different superscripts differ significantly (p<0.05). SC=Scrotal circumference, SVs=Seminal vesicles, SEM=Standard error of mean

**Table-4:** Distribution of SC and SVs as per mean and 1SD above mean within each dentition age groups of buffalo bull.

Dentition age	Number of bulls with scrotal circumference and seminal vesicle			

	≥Mean value	%	Above mean+1SD	%
Dentition-I				
SC	13/27	48.15	24/27	88.89
SVs	13/27	48.15	21/27	77.78
Dentition-II				
SC	13/24	54.17	21/24	87.50
SVs	12/24	50.00	20/24	83.33
Dentition-III				
SC	4/08	50.00	6/08	75.00
SVs	5/08	62.50	6/08	75.00

SC=Scrotal circumference, SVs=Seminal vesicles, 1SD=One Standard deviation

**Table-5:** Mean±SEM of SC (cm), total SV weight (g), plasma testosterone concentration (ng/ml), SV protein (mg/2 ml), SV fructose (mg/2 ml), and SV citric acid (mg/2 ml) in three dentition age groups in male buffalo

Parameters	Dentition-I	Dentition-II	Dentition-III
N	27	24	08
SC	18.3±0.38^c^	21.7±0.40^b^	24.6±0.5^a^
Testosterone	0.05±0.01^b^	0.16±0.04^b^	0.65±0.16^a^
Total SV weight	5.04±0.48^c^	10.45±0.95^b^	15.57±1.90^a^
SV proteins	5.19±0.96^c^	13.51±1.35^b^	21.54±2.38^a^
SV fructose	0.78±0.27^b^	4.85±0.69^a^	6.49±1.57^a^
SV citric acid*	0.68±0.12^c^	2.27±0.36^b^	3.55±0.76^a^

* N=26, 22, and 8 observations respectively. Different superscripts differ significantly (p<0.05). SC=*Scrotal circumference, SVs=Seminal vesicles, SEM=Standard error of mean*

### Effect of SV weight and age on secretion

Secretion of SVs (protein, fructose, and citric acid) showed significant positive correlation with SC, testosterone, and total SVs weight and concentration varied as per increasing SV weight and dentition age (Tables-3 and 5). Protein concentration registered significant (p<0.05) increasing trend from lower SVs weight Group I to higher weight Group III (Table-3). Similar to SVs protein, fructose concentration also showed ascending trend from small-to-large SV weight groups with a significantly (p<0.05) higher value in Group III compared to Groups II and I; however, the difference between Groups I and II was not significant. While citric acid concentration in SVs weight Group -III showed significantly (P<0.05) higher value compared to SVs weight Groups –II and -I, but its concentration revealed declining trend (P>0.05) from SVs weight of Groups -I to -II. Interestingly, similar to SVs weight groups, SVs protein and fructose also showed ascending increase from lower to higher dentition age groups, but the difference in fructose concentration between dentition-II and -III was not significant (p>0.05). However, citric acid concentration showed significant (p<0.05) increase from lower to higher dentition age groups contrary to SV weight groups (Table-5). Intraassay coefficient of variation of SVs protein and fructose concentration was 16.87% and 11.04%, respectively.

### Sequence analysis

On sequence analysis, the full-length, *B. bubalis* (Water buffalo) sequenced BSP1 gene (GenBank Acc.no. KR703587) showed an identity of 99%, 93%, and 88% with BSP1 of *B. bubalis* (Murrah: GenBank Acc.no. KM114211.1), *B. taurus* (cattle: GenBank Acc.no. NM_001001145.1), and *Ovis aries* (sheep: GenBank Acc.no.NM_001143665.2), respectively, while it also showed 96% identity with *B. taurus* (cattle) BSP3 gene (GenBank Acc.no. NM_174840.1). On the other hand, BSP3 nucleotide sequence analysis (GenBank Acc.no. KR814819.1) showed an identity of 98% and 86% with BSP3 gene of *B. bubalis* (GenBank Acc.no.JX280019.1) and *B. taurus* (GenBank Acc. no. NM_174840.1, respectively. Furthermore, buffalo BSP5 nucleotide sequence analysis (GenBank Acc.no. KR869821.1) indicated 95% identity with BSP5 gene of *B. taurus* (GenBank Acc.no. NM_174842.2).

### Phylogenetic analysis

**Figure-5:**
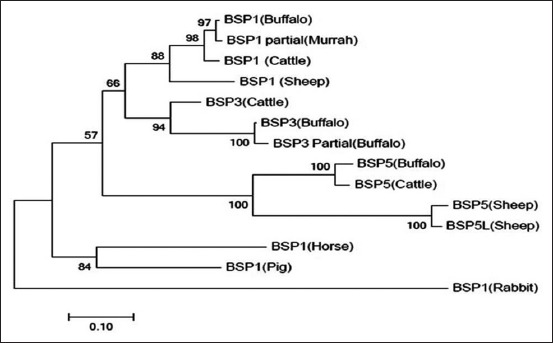
Phylogram depicting the buffalo BSP (BSP1, BSP3 and BSP5) nucleotide evolutionary relationship with BSPs of other species. The length of branch depicting divergence. Numbers on branches depicts bootstrapping (%) support from 1000 replications.

Phylogenetic analysis (Figure-5) of nucleotide sequence of the three BSP subgroups revealed clustering of the BSP1,3, and 5 into three distinct groups. BSP1 of the horse, pig, and rabbit clustered away from the ruminant groups. The ruminant BSP1, 3, and 5 clustered with a bootstrapping of 88%, 94%, and 100%. Phylogram also indicated common evolutionary relationship among cattle (*B. taurus*, 2n=60) and sheep (*Ovis aries*, 2n=54), and BSP1 of horse (*Equus caballus*, 2n=64) was found more closely related to pig (*Sus scrofa*: 2n=38) followed by outgroup coprophagus rabbit (*Oryctolagus cuniculus*, 2n=44).

## Discussion

A significant positive correlation of SC with that of testosterone, SV weight, and its secretion (protein, fructose, and citric acid) was observed to be considered as a predictive model for grading buffalo bulls in particular. These parameters were derived from apparently healthy SVs without any inflammatory condition which otherwise might adversely affect SV function and sperm motility [16]. In the present study, varying tubular length with uni- or bi-lateral budding and lobulation patterns of shape could be due to increasing age, progressing SV weights, and testosterone levels as SV growth and function are androgen dependent [11]. Our findings corroborate with the findings in immature and mature buffalo bulls [17] and morphology during pre-pubertal, pubertal, and post-pubertal stage [18] in male buffalo. Furthermore, findings of SV histology are also comparable with reports in buffalo [17].

Nutritional status is the prime determinant of reproductive fitness for all life forms [19]. In the present study, macronutrients (plasma protein, glucose, and cholesterol) of sacrificed male buffaloes indicated near uniform nutritional status comparable with that of Lapitan *et al*. [20] and Sharma *et al*. [21].

As per our findings, linear regression fit equation showed 1 g increase in SV weight (an indicator, of semen volume) for every 1.395 cm increase in SC (Figure-4). In beef bull, Berry *et al*. [22] also recorded a significant correlation between SC and SVs. In addition, SC and testicular volume reflect sperm production capacity of bulls [23]. We found a significant increase in SC from lower to higher dentition age, which is comparable to the findings in Murrah buffalo [2]. Furthermore, SC of Murrah buffalo bull was reported lesser probably due to slow growth similar to our findings compared to Holstein bull of the same age [2]. Thus, selection of bulls for optimum SC can have a greater impact on future reproductive potential of progenies.

Testosterone also showed a significant positive correlation with SC and SVs weight, and thus, estimation of circulating testosterone can be used as a measurable marker of organ (SC and SVs) weight, function, and libido. In male buffaloes, Shinde [15] reported a significant correlation between SV fluid testosterone and SV weight contrary to the present findings. The present studies also demonstrated ascending increase in testosterone concentration as per SV weight and dentition age. Interestingly, comparable to dentition age, Habeeb *et al*. [24] also reported progressive testosterone increase from birth to puberty in buffalo calf. In young and adult male buffalo, Gunarajasingam *et al*. [25] reported wide variation in testosterone (0.2-2.7 ng/ml) possibly due to age, diurnal, and episodic pattern of secretion. In rat, Higgins and Burchell [11] reported that testosterone regulates mRNA population of SV resulting in enhanced growth and function. Thus, proving the hypothesis that testosterone level (also predicted by SC size) could be considered as an indicator of male reproductive performance, and thus, its estimation can be useful in grading and selection.

In addition, size/weight and function of SVs could be predicted by measuring SC and testosterone as SVs registered a significant correlation with SC and plasma testosterone. Contrary to the present observation, a significant positive correlation was reported between SV weight and SV fluid testosterone in male buffalo [15]. The present study also recorded a significant effect of dentition age on SVs weight comparable to findings in water buffalo [26], while in another report, SV weight was lower in immature and mature buffalo bulls [17] possibly due to less number of observations and due to varying age and nutritional status which govern growth.

Interestingly, a significant positive correlation of SC, SVs, and testosterone was established with SVs protein, fructose, and citric acid under uniform macronutrient status (Table-1) as underfeeding of young calf declines responsiveness of SVs to testosterone [27]. The present finding reported a significant increase in SVs protein. However, earlier study reported non-significant increase as per SV weight and testicular weight groups [12,28], respectively, probably due to varying age and SV weight. Furthermore, both fructose and citric acid were significantly (p>0.05) higher in higher SV weight and dentition age groups probably due to larger SC and in turn increased testosterone level and SV weight. In bulls, Hay *et al*. [29] also reported a significant correlation between growth and secretory activity of SVs. Similarly, Samuels and Harding [30] also reported the effect of age on the level of fructose and citric acid in SV tissue of bulls. Thus, estimation of this constituent can be used to detect hypofunction of SVs caused by age, management, or inflammation which influences fertility by altering sperm chromatin stability and motility [16]. These observations supported the hypothesis that larger SC could predict the testosterone, SV weight, and its secretory function modulating semen quality.

Furthermore, BSP 1, 3, and 5 genes were sequenced in SV tissue and analyzed and evaluated for their phylogenetic relationship (Figure-5). In buffalo SVs, Barik [31] and Kutty [32] reported partial sequence of BSP1 and BSP3, respectively, which were comparable to full-length sequence of the present findings. BSP1 of buffalo also showed identity with cattle and sheep in spite of variability of chromosome number, which is a dynamic feature of eukaryotic evolution [33]. Interestingly, buffalo had more closer identity with cattle because of chromosomal homology [34]. Our observation of phylogenetic analysis showed the clustering of buffalo BSP1 with cattle and sheep away from monogastric (horse and pig), while rabbit formed distinct out pocket. Interestingly, the present findings indicated closer identity between BSP1 and 3 compared to BSP5 in concordance to the reports of Jois *et al*. [14] in cattle. BSP1 of horse and pig clustered away from the BSP1, 3, and 5 of large and small ruminants. Interestingly, high bootstrapping values observed in the present report further proved the use of gene in classifying ruminant and non-ruminant into different classes. The coprophagus rabbit BSP1 as an outgroup showed close phylogenetic relationship with BSP1of monogastric animals (pig and horse) indicating importance of rabbit BSP1in the evolution of BSPs with changing physiological need, environment and speciation. [35]. Hence, BSPs and other major proteins along with parameters of present finding can be useful as an indicator of fertility for grading and selection of outstanding breeding bull.

## Conclusion

The present findings established a significant positive correlation of SC with that of other fertility parameters related to SV weight, its secretions: Fructose, citric acid, and protein (inclusive of BSPs sequenced full length), and testosterone. Therefore, the present integrated approach along with certain semen quality attributes reflecting epididymis function could be used as a predictive fertility marker for grading and selection of breeding bulls and their progenies to develop outstanding bull mother farm.

## Recommendations

To further corroborate the present findings, controlled experiment on larger number of males involving age, season, body weight, breed, and puberty of progenies maintained under optimum nutrition along with estimation of BSP proteins, hormones, and seminal parameters involving testicular, SVs, and epididymal attributes is warranted to device prediction model for selection of outstanding sire and their progenies.

## Author’s Contributions

SM designed the experiment, collected SV and blood samples from sacrificed male buffalo, processed samples for RNA extraction and cDNA preparation, PCR and purification of PCR product for sequencing, biochemical analysis of blood plasma and SV flushed fluid, review and writing of manuscript, AK designed primers and post-sequencing editing, RS histomorphological screening, and interpretation, MS provided expertise/facilities and in editing of manuscript, GS analysis of plasma testosterone and editing, GVPPSRK contributed to sequence and phylogenic analysis and interpretation while MRV provided statistical analysis using SAS 9.2. All authors read and approved the final manuscript.
